# ENSO’s far reaching connection to Indian cold waves

**DOI:** 10.1038/srep37657

**Published:** 2016-11-23

**Authors:** J. V. Ratnam, Swadhin K. Behera, H. Annamalai, Satyaban B. Ratna, M. Rajeevan, Toshio Yamagata

**Affiliations:** 1 Application Laboratory, Japan Agency for Marine-Earth Science and Technology, Yokohama Japan; 2 International Pacific Research Center and Department of Oceanography, University of Hawaii, USA; 3 Ministry of Earth Sciences, New Delhi, India

## Abstract

During boreal winters, cold waves over India are primarily due to transport of cold air from higher latitudes. However, the processes associated with these cold waves are not yet clearly understood. Here by diagnosing a suite of datasets, we explore the mechanisms leading to the development and maintenance of these cold waves. Two types of cold waves are identified based on observed minimum surface temperature and statistical analysis. The first type (TYPE1), also the dominant one, depicts colder than normal temperatures covering most parts of the country while the second type (TYPE2) is more regional, with significant cold temperatures only noticeable over northwest India. Quite interestingly the first (second) type is associated with La Niña (El Niño) like conditions, suggesting that both phases of ENSO provide a favorable background for the occurrence of cold waves over India. During TYPE1 cold wave events, a low-level cyclonic anomaly generated over the Indian region as an atmospheric response to the equatorial convective anomalies is seen advecting cold temperatures into India and maintaining the cold waves. In TYPE2 cold waves, a cyclonic anomaly generated over west India anomalously brings cold winds to northwest India causing cold waves only in those parts.

Cold waves that occur during boreal winter months from November to February, exert considerable stress to the people of northern India. During cold wave episodes, a drop of more than 4 °C is observed in minimum temperatures, and these episodes generally persist for 3–5 days^1^ and their occurrence peaks in the month of January^1^. Earlier studies^2^ showed that cold waves occur mostly due to the intrusion of cold air from northern latitudes into the northwestern parts of India. The cold wave conditions over the northern parts of India are often associated with the passage of western disturbances^2^. Western disturbances, which manifest as an eastward moving well marked troughs in the upper tropospheric westerlies north of 20°N and often seen extending to the lower troposphere^2^,^3^,^4^, transport cold air from northern latitudes into India. There are also few instances of occurrence of cold waves due to a low pressure system over the North Arabian Sea^2^. In these cases, the easterlies to the north of the low pressure system transport cold air from higher latitudes.

The cold waves are known to increase mortality rate owing to the socio-economic conditions of people of the northern parts of India. For example, the cold wave that occurred in January 2003 resulted in death of about 900 people^5^. During 1978–1999, a total number of 3264 deaths were reported due to cold waves in the northern parts of India^6^. The cold waves also affect the Rabi crops, crops that are sown in winter and harvested in the following spring, of the northern regions of India^7^,^8^. A survey on the impact of cold wave on the Rabi crops showed that the economic losses were to the tune of 6230 million Indian rupees in the state of Rajasthan during 2005–2006 Rabi season alone^7^.

Though it is known that cold waves are associated with the western disturbances, to our knowledge, there are no systematic studies to understand the origin and sustenance of these disturbances. Using observed and reanalysis datasets, we made an effort to understand the processes causing the cold waves and the processes maintaining them.

## Results

### Minimum Temperature Anomalies

**Figure 1:**
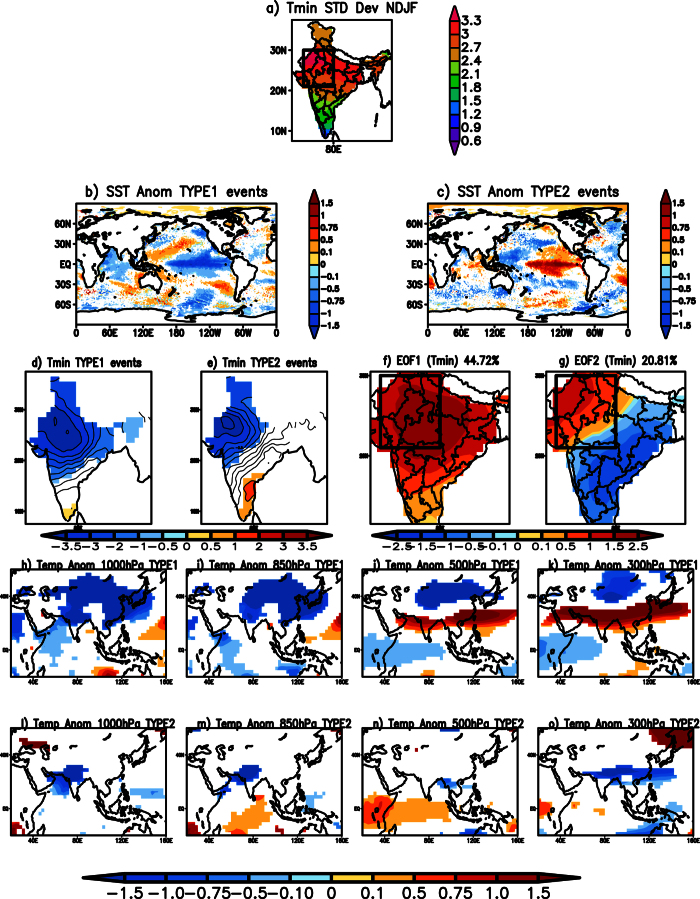
(**a**) Standard deviation of the Tmin (°C) from 1^st^ Nov to 28^th^ Feb over the period 1982 to 2013. (**b**) Spatial distribution of significant SST (°C) anomalies during TYPE1 events. (**c**) same as (**b**) but for TYPE2 events. (**d**) Significant Tmin (°C) anomalies associated with TYPE1 events (**e**) same as (**d**) but associated with TYPE2 events. (**f**) The first mode of EOF of Tmin anomalies. (**g**) The second mode of EOF of Tmin anomalies. (**h–k**) Significant air temperature anomalies (°C) at 1000 hPa, 850 hPa, 500 hPa and 300 hPa respectively during TYPE1 events. (**l–o**) Significant air temperature anomalies (°C) at 1000 hPa, 850 hPa, 500 hPa and 300 hPa during TYPE2 cold wave events. Significance is at 99% using two-tailed Student’s t-test. The rectangular box in (**a**), (**f**) and (**g**) represents the region used to identify the cold wave events over India. (Figure was created using a free software Grid Analysis and Display System (GrADS) version 2.1.a3 (http://cola.gmu.edu/grads/downloads.php)).

As a measure of intensity of cold waves, we examine statistics of daily minimum temperature (T_min_ hereafter). Figure 1a shows the standard deviation of T_min_ observed over the Indian landmass for the boreal winter months of November to February during 1982 to 2013. The standard deviation shows marked variability (upwards of 3 °C) over northwest India. To identify cold wave events in historical data, based on coherent fluctuations in daily minimum surface temperature (T_min_), we define an index by taking an area average of T_min_ anomalies over the region 71°E-80°E; 21°N-30°N (Fig. 1a). It may be noted here that the results are not much changed with a slight variation in the chosen area. A cold wave event is identified when the defined index in a chosen day is less than one standard deviation, and the anomalies persist for at least 4 days. Furthermore, the standard criterion adopted by the India Meteorological Department (IMD) for defining a cold wave is also considered. The procedure is given in the methods section.

Based on the criteria, twenty-nine events were identified (Tables 1 and 2) with a propensity of maximum number of events occurring in the months of January (12) and February (10) (Tables 1 and 2). The dates of the events agree with the dates of IMD. To understand if these cold events over India are associated with large-scale changes that could be used as sources of predictability, we compared the period in which the cold wave events occurred with the historical NINO3.4 (5°N-5°S; 120°-170°W) index maintained by the Climate Prediction Center, National Center for Environmental Predication (NCEP), USA. Out of the identified 29 events, 21 (8) events occurred during La Niña (El Niño) years suggesting that both phases of ENSO favor the occurrence of cold wave events over India. Of the 21 events, which occurred during La Niña years, the NINO3.4 area-averaged SST anomalies for four events (events 3, 4, 7 and 21 in Table 1) during December-February were −0.3, −0.3, −0.4 and −0.4 °C- values just above the threshold temperature of −0.5 °C criteria used by the NCEP for identifying a La Niña event. Similarly, one event (event 6 in Table 2), from the events associated with El Niño, shows warm temperatures of 0.4 °C over the NINO3.4 region for the season, a value that is just below the criteria of 0.5 °C used by the NCEP for identifying an El Niño event. Not surprisingly, SST anomalies composited corresponding to the twenty-one events in La Niña years show cooler than normal SST anomalies along the equatorial Pacific extending from the central to eastern Pacific with concurrent warmer than normal SST anomalies in the equatorial west Pacific (Fig. 1b). Similarly, SST anomalies composited for the eight events corresponding to El Niño years, show significant positive SST anomalies in the equatorial Pacific extending from central to eastern Pacific, with cooler than normal SST anomalies in the west Pacific (Fig. 1c). Hereafter, we term cold events that occurred during La Niña (El Niño) as TYPE1 events (TYPE2 events). Furthermore, compositing T_min_ anomalies for TYPE1 events show significant reduction in T_min_ over most parts of India (Fig. 1d). On the other hand, for TYPE2 events significant reduction in T_min_ is confined mostly to northwest India, with positive anomalies along the east coast of India (Fig. 1e).

In order to objectively separate the modes of variation we have adopted an empirical orthogonal function (EOF) analysis over the region 70°E-90°E; 12°N-30°N. Figure 1f shows the first EOF mode that explains 44.72% of the total variance. This mode has large loadings over the region of high standard deviation of the T_min_ temperatures (Fig. 1a) and has a pattern similar to the spatial distribution of T_min_ anomalies during TYPE1 cold wave events (Fig. 1d). Furthermore, correlation between the T_min_ anomalies averaged over the region 71°E-80°E; 21°N-30°N with this mode’s principal component is 0.91. The second mode (EOF2) (Fig. 1g) explains about 20.81% variability of the total variance and shows a dipole structure, similar to TYPE2 cold wave events (Fig. 1e), with opposite phases over the north-west India and along the east coast of India. The correlation of its principal component with the T_min_ anomalies averaged over the region 71°E-80°E; 21°N-30°N is only 0.385. Thus, the EOF analysis justifies our procedure of separating the observed cold wave events into TYPE1 and TYPE2.

We plotted temperature anomalies at different levels to see the vertical extent of the cold anomalies. During TYPE1 events, composite air temperature anomalies at 1000 hPa, 850 hPa, 500 hPa and 300 hPa levels (Fig. 1h–k) show that over the northern parts of India colder than normal air is confined to about 850 hPa while warmer air prevails at 500 hPa and above. This is in contrast to the presence of cold air throughout the troposphere over northern China (Fig. 1h–k). Therefore, over India, low-level winds would be an effective agent in advecting cold air from north. During TYPE2 events the colder than normal temperatures are confined to the northwestern parts of India in the lower troposphere (Fig. 1l–m). The temperatures over India are not significant at 500 hPa (Fig. 1n) though significant anomalously cold temperatures are seen at 300 hPa over the Indian latitudes (Fig. 1o) during the TYPE2 events.

We analyze the causes of the negative minimum temperature anomalies over the Indian region in the following sections.

### TYPE1 Cold waves

**Figure 2:**
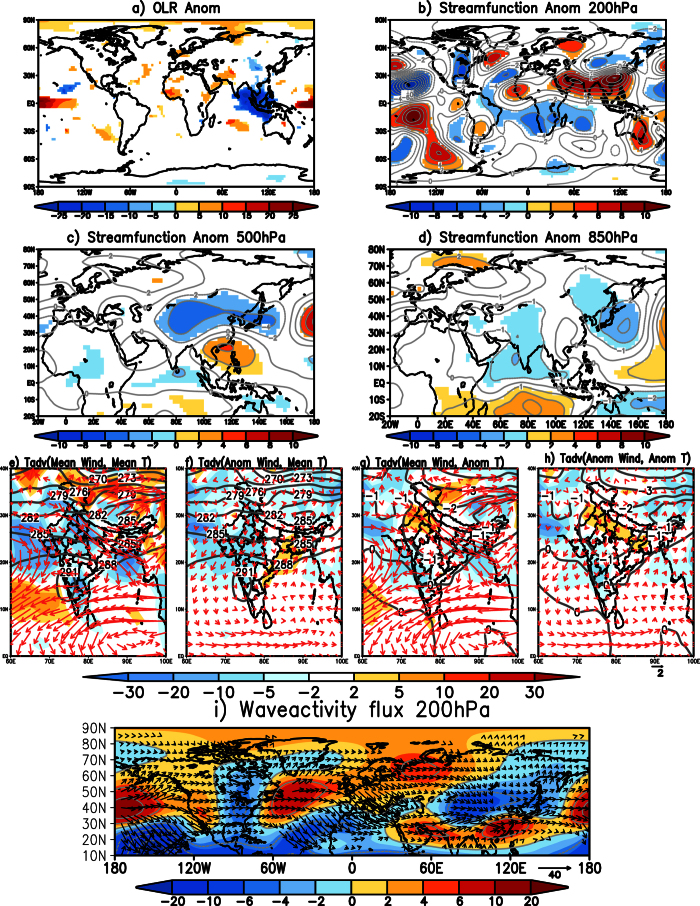
(**a**) Significant OLR (W/m^2^) anomalies of the composite of TYPE1 cold wave events. (**b,c** and **d**) same as (**a**) but for significant eddy streamfunction (×10^6^ m^2^ s^−1^; shaded) at 200 hPa, 500 hPa and 850 hPa levels respectively. (**e**) Horizontal temperature advection (shaded; ×10^−6^ °Ks^−1^) of mean temperature by mean winds at 850 hPa during TYPE1 events. Contours represent mean temperatures and mean wind at 850 hPa is shown by vectors (**f**) same as (**e**) but advection of mean temperature by anomalous wind (shaded). Mean temperature (contour) and anomalous winds (vectors) are also shown (**g**) same as (**e**) but advection of anomalous temperature by mean winds (shaded). 850 hPa temperature anomalies are shown as contours and mean winds as vectors (**h**) same as (**e**) but advection of anomalous temperature by anomalous winds (shaded). Contours represent temperature anomalies and vectors represent anomalous winds at 850 hPa (**i**) Significant wave activity flux anomalies at 200 hPa (vector; either zonal or meridional component is significant) and the streamfunction anomalies (shaded) for TYPE1 cold wave events. Significance is at 99% using two-tailed Student’s t-test. (Figure was created using a free software Grid Analysis and Display System (GrADS) version 2.1.a3 (http://cola.gmu.edu/grads/downloads.php)).

The TYPE1 events exert considerable stress over most parts of the country. Here we investigate the mechanisms and processes associated with these types of cold waves. Figure 2a shows the spatial distribution of composite outgoing longwave radiation (OLR)^9^ anomalies, a proxy to tropical precipitation anomalies. In response to colder than normal SST anomalies, positive OLR anomalies (indicative of less cloud cover) are observed over the equatorial central Pacific while negative OLR anomalies (indicative of excess cloud cover and more rainfall) are noted from west of the Philippines to the east coast of India. Over northwest India and extending into Pakistan, prevailing colder than normal air temperature in the boundary layer (Fig. 1h–k) enhances the stability and thus limit convection development resulting in positive OLR anomalies there (Fig. 2a).

To understand if a remote teleconnection was the cause of the TYPE1 cold waves, we plotted composite of 200 hPa eddy (zonal mean removed) streamfunction anomalies (Fig. 2b). Prominent features evident in the composite (Fig. 2b) and which could have contributed to the TYPE1 cold wave events over India can be divided into two parts. i) A region of anomalous positive anomalies over the Ural-Siberia region and a wave train originating from there and extending to South China with a trough over most parts of China and a ridge over South China and the northeastern parts of India and, ii) A region of anomalous anticyclone over the Indian landmass. The anomalous wave pattern from the Ural-Siberia region and extending to South China seen in Fig. 2b, is often observed during cold surge events over East Asia^10^. The wave train from the Ural-Siberia region was the cause of the cooler than normal temperatures observed over China during the events (Fig. 1h–k). The calculation of waveactivity flux anomalies^11^ at 200 hPa (Fig. 2i) also shows fluxes from the region of anomalous positive anomalies over the Ural-Siberia region to the South China Sea. The waveactivity flux anomalies also show that the anomalous positive anomalies over the Ural-Siberia region may not necessarily be linked to the climate conditions over the equatorial Pacific. Nevertheless, the cold waves though not directly linked to cooler SST anomalies in the Pacific, are anchored by the low level cyclonic anomaly generated by the anomalous Matsuno-Gill^12^,^13^ response to the anomalous maritime convections, as discussed later. This feature, that helps in transporting cold temperatures from higher latitudes into Indian latitudes and sustain the cold waves, is also seen clearly in Fig. 2b as an anticyclonic anomaly at 200 hPa over the Indian latitudes.

The anomalous trough over China and the anomalous ridge over South China is seen extending from 500 hPa to 200 hPa level (Fig. 2c). At 850 hPa (Fig. 2d), the anomalous ridge over the Ural-Siberia region and the associated trough is seen covering the Indo-China region causing cooler than normal temperatures over the region (Fig. 1i). The trough that extends throughout the troposphere results in colder than normal temperatures over China (Fig. 1h–k). Also, at 850 hPa (Fig. 2d) a pair of anomalous cyclones is seen to straddle the equator in the Indian Ocean as the Matsuno-Gill response to the equatorial convection in the region extending from the Bay of Bengal to the west of Philippines (Fig. 2a). The atmospheric response to equatorial convection, however, is baroclinic in nature and is seen as an anomalous anticyclone at 200 hPa (Fig. 2b) over the Indian landmass and the surrounding regions. The north-south orientation of the cyclonic anomaly over north India is favorable for advection of cold air from higher latitudes into India.

We calculated the 850 hPa horizontal advection of mean observed temperature by mean observed winds (Fig. 2e) to investigate if the cold waves over India are caused by horizontal advection of cold air from higher latitudes. During TYPE1 type events, northwesterly winds are seen advecting cold temperatures from higher latitudes into the Indian subcontinent resulting in reduction of the temperatures over the Indian landmass (Fig. 2e). To understand the role of the anomalous cyclone generated as a response to the tropical heating in the maintenance of the cold waves, we examined the contribution of the advection of mean observed temperature by the anomalous winds (Fig. 2f), the contribution of the advection of the anomalous temperature by the mean observed winds (Fig. 2g) and the advection of the anomalous temperature by the anomalous winds (Fig. 2h). Comparing the contribution of the three terms (Fig. 2f–h), it is seen that the advection of the mean observed temperature by the anomalous cyclonic response (Fig. 2f) largely explains the cold air advection over large parts of India. This term (Fig. 2f) has a higher contribution compared to any other terms (Fig. 2g–h). The anomalous cyclonic winds advect warmer temperatures from the equatorial region resulting in higher than normal temperature over the east coast of India (Fig. 2f). The effect of the cold advection by the anomalous winds is reduced over the northern parts of India by warm advection of the anomalous temperature by the mean winds (Fig. 2g) and also by the advection of the anomalous temperature by the anomalous winds (Fig. 2h). Both the advection of the anomalous temperature by the mean observed wind (Fig. 2g) and by the anomalous wind (Fig. 2h) are seen to bring cooler temperatures to the head Bay of Bengal and also to the western parts of India.

**Figure 3:**
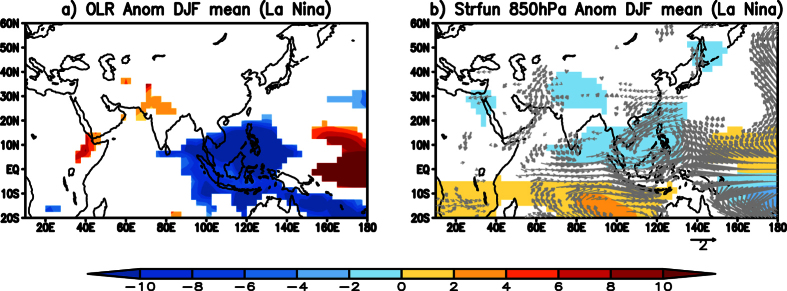
(**a**) Significant OLR anomalies composited over Dec–Feb months during the La Niña years over the period 1982 to 2013. (**b**) same as (**a**) but is for significant eddy streamfunction (×10^6^ m^2^ s^−1^) anomalies at 850 hPa and significant wind anomalies (vectors; either zonal or meridional component is significant). Significance is at 90% using two-tailed Student’s t-test. (Figure was created using a free software Grid Analysis and Display System (GrADS) version 2.1.a3 (http://cola.gmu.edu/grads/downloads.php)).

The low level cyclonic anomaly observed over India during the TYPE1 cold wave events (Fig. 2d) may be partly attributed to the seasonal mean equatorial cyclonic anomaly observed during La Niña events. Figure 3 shows composite OLR anomalies, 850 hPa eddy streamfunction (shaded) and 850 hPa wind (vector) anomalies constructed by averaging December-February monthly anomalies during La Niña years over the period 1982 to 2013. In response to a spatially coherent negative OLR anomaly noted over the Maritime Continent and the west Pacific regions (Fig. 3a), a pair of cyclones straddling the equator is seen (Fig. 3b) over the Indian Ocean. The poleward flank of the Northern Hemisphere part of the cyclonic anomalies (Fig. 3b) is favorable to advect cold temperatures from higher latitudes into the Indian region during La Niña years.

**Figure 4:**
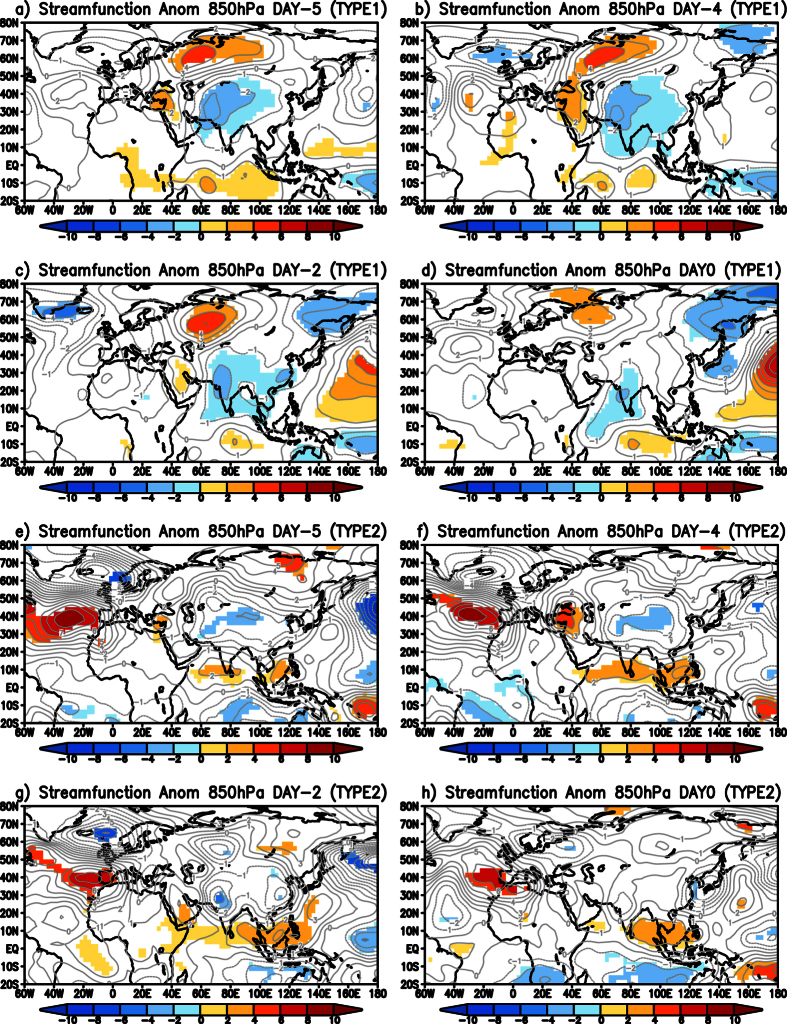
(**a**) Composite of significant eddy streamfunction (×10^6^ m^2^ s^−1^) anomalies at 850 hPa five days before TYPE1 events (DAY-5). (**b,c** and **d**) same as (**a**) but four days (DAY-4) before, two days (DAY-2) before and on the day (DAY0) respectively of the TYPE1 event. (**e–h**) same as (**a–d**) but for TYPE2 cold wave events. Significance is at 90% using two-tailed Student’s t-test. (Figure was created using a free software Grid Analysis and Display System (GrADS) version 2.1.a3 (http://cola.gmu.edu/grads/downloads.php)).

We composited the 850 hPa eddy streamfunction anomalies of all the TYPE1 cold wave events from 5 days (DAY-5) before the event to the day the event started (DAY0), to understand the initiation of the cold wave events over India and presented the results in Fig. 4. Day0 is considered the day when the events started. Five days before the TYPE1 cold wave events occurred over India (DAY-5) (Fig. 4a), anomalous high in the higher latitudes and the associated wave with anomalous trough over China and over India are oriented north to south. The trough has a maximum to the northwest of India (Fig. 4a). The anomalous trough is seen moving eastward from DAY-4 (Fig. 4b–d) and is seen covering whole of the Indian landmass. The maxima in the cyclonic anomaly is over India from DAY-2, two days before the events started (Fig. 4c). On DAY0, the anomalous wave is oriented such that an anomalous cyclone is seen anchored to Indo-China region. The anomalous cyclone to the south of equator in the Indian Ocean is seen from DAY-5 to DAY0 (Fig. 4a–d), however it intensifies from DAY-2 (Fig. 4c). As the TYPE1 events occurred during La Niña type of SST distribution in the equatorial Pacific, the spatial distribution of the equatorial convection in those years provides the required background for the anomalous Matsuno-Gill response (Fig. 3). The daily variations in the anomalous diabatic heating influence the intensity and location of the anomalous Matsuno-Gill response. Our study shows that the anomalous Matsuno-Gill response intensifies two days before the cold wave conditions over the Indian Landmass and anchors the wave to the Indian region. Most notably, the cyclonic anomaly over India and its corresponding pair in the equatorial South Indian Ocean persist through the life period of the cold wave event (Fig. 2d).

The anomalous high pressure over the Ural-Siberia region (Fig. 2b–d) seen during the TYPE1 cold wave events over India, is sometimes linked to an atmospheric blocking event. Here, we investigate if all the atmospheric blocking events over the region could be linked to TYPE1 cold waves over India. We identified atmospheric blocking events of duration of four or more days, over the longitudes 20°W to 120°E, and over the period NDJF 1982/83 to NDJF 2012/13, using a blocking index^14^. We applied 5-day running mean to the 500 hPa geopotential height field before applying the index. We classified the atmospheric blocking events into two classes: (i) those which occurred during La Niña years, and (ii) El Niño and positive neutral years, associated with positive SST anomalies in the NINO3.4 region though weaker in magnitude. We identified a total of 76 blocking events during La Niña years and 69 events during other El Niño and neutral years.

**Figure 5:**
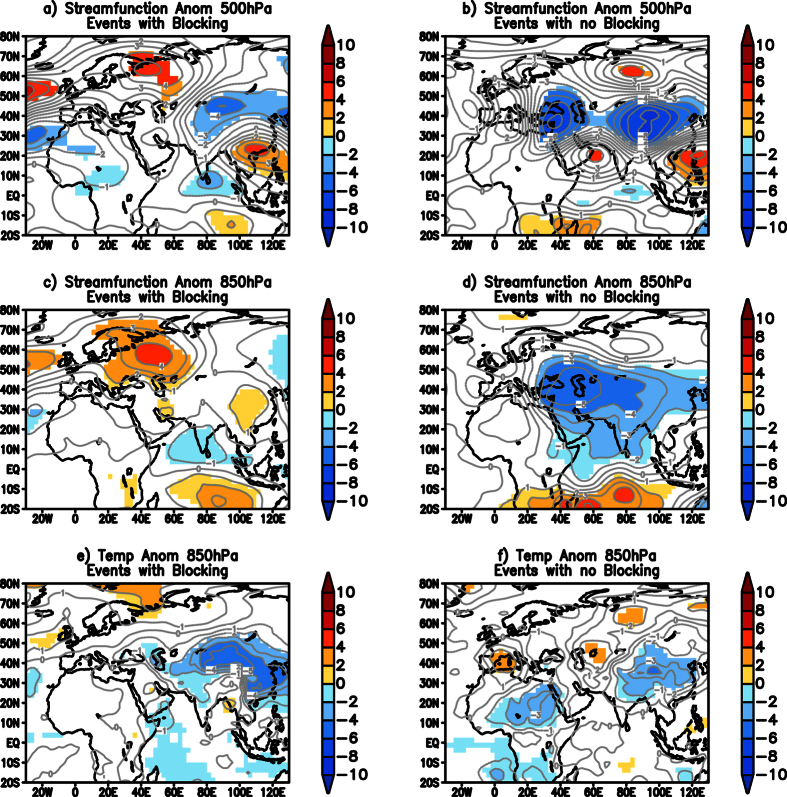
Composite of significant eddy streamfunction (×10^6^ m^2^ s^−1^) anomalies of TYPE1 events associated with blocking in the Ural-Siberia region at (**a**) 500 hPa and (**c**) 850 hPa. (**b** and **d**) are same as (**a** and **c**) respectively but for TYPE1 events not associated with blocking over Ural-Siberia region. (**e**) is same as (**c**) but for temperature anomalies (oC). (**f**) same as (**d**) but for temperature anomalies. Significance is at 99% using two-tailed Student’s t-test. (Figure was created using a free software Grid Analysis and Display System (GrADS) version 2.1.a3 (http://cola.gmu.edu/grads/downloads.php)).

On comparing the 76 blocking events during the La Niña years to the 21 TYPE1 cold wave events, we found that 13 of the TYPE1 cold wave events were associated with atmospheric blocking events over the Ural-Siberia region. These 13 TYPE1 cold events, and the remaining 8 TYPE1 events not associated with atmospheric blocking are composited separately and presented in Fig. 5. The 500 hPa eddy streamfunction anomalies of both the composites show positive anomalies over the Ural-Siberia region (Fig. 5a,b) and a wave train from there and reaching South China with an anomalous trough over China and over the latitudes to the north of India, though the magnitude of the ridge is stronger in the composite associated with the blocking events (Fig. 5a). The ridge over the Ural-Siberia region is also significant at 850 hPa in the composite of the events associated with atmospheric blocking (Fig. 5c), while the positive anomalies are insignificant in the other composite not associated with blocking (Fig. 5d). Cyclonic anomalies over India are seen at 850 hPa in both the composites (Fig. 5c and d). A composite of 850 hPa temperature anomalies shows anomalous cool temperatures over the Indo-China region in both the composites (Fig. 5e and f), though the extent and magnitude of anomalous cool temperature are higher in the composite of events associated with blocking over the Ural-Siberia region. The above analysis shows that the cold waves over India can occur with or without blocking events over the Ural-Siberia region but what is important is the presence of anomalous low temperatures over the Indo-China region which can be advected into the Indian latitudes by the anomalous cyclonic circulation over India.

**Figure 6:**
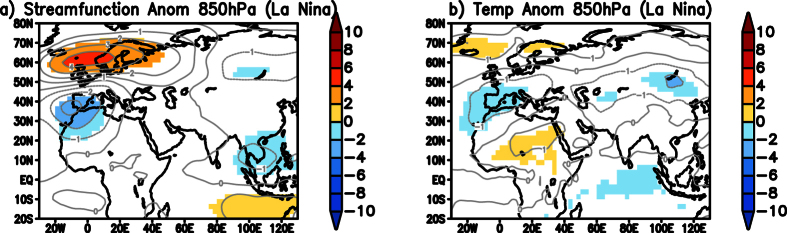
Composite of significant 850 hPa eddy streamfunction (×10^6^ m^2^ s^−1^) anomalies of blocking events during La Niña years over higher latitudes not associated with TYPE1 cold wave events over India. Significance is at 90% using two-tailed Student’s t-test. (Figure was created using a free software Grid Analysis and Display System (GrADS) version 2.1.a3 (http://cola.gmu.edu/grads/downloads.php)).

In a separate analysis, 63 blocking events, observed during La Niña years but not associated with cold wave conditions over India, are composited (Fig. 6) to understand why those blocking events over higher latitudes during La Niña years did not lead to cold wave conditions over India. In the 850 hPa eddy streamfunction composite, anomalous high can be seen over the Ural-Siberia region (Fig. 6a) with an anomalous low over China. The anomalous low over China is seen causing low temperatures over the region (Fig. 6b). However, these anomalous cold temperatures are not significant toward latitudes closer to India. Also, the anomalous cyclonic circulation over India is not prominent in the composite (Fig. 6a). Therefore, significant cold temperatures in the latitudes closer to northern India and the low-level cyclonic circulation anomalies over India are important factors to favor advection of cold temperatures into India leading to cold wave conditions. Hence, blocking over the Ural-Siberia region is not a necessary condition to favor TYPE1 cold waves over India.

The above analysis shows that the TYPE1 cold waves are seen to be associated with a northwest-southeast oriented wave train from the Ural-Siberia region to South China with a cyclonic anomaly over the Indo-China region. The northwest-southeast oriented wave train results in cooler than normal temperatures over China in the latitudes closer to the northern parts of India. An anomalous low level trough as a part of an anomalous wave train from the Ural-Siberia region is seen anchored to the Indian latitudes by the anomalous Matsuno-Gill response to the anomalous equatorial tropical convection from 2 days before the start of the event and is seen persisting throughout the life of the cold wave. The anomalous trough is oriented such as to encourage anomalous transport of cold temperatures from higher latitudes into India, thus resulting in TYPE1 cold waves over India.

### TYPE2 Cold waves

Composite of T_min_ anomalies during TYPE2 cold wave events shows a significant negative anomaly confined to northwest India (Fig. 1e). We examined low-level temperature anomalies and 200 hPa eddy streamfunction anomalies of the individual events to understand the physical processes causing the TYPE2 cold waves. Of the eight identified events, six events had a similar distribution of temperature and eddy streamfunction anomalies and the other two events, 5 and 7 (Table 2), had 200 hPa wave pattern with a phase slightly different from the other six events. Therefore, we composited the six events to understand the characteristics of TYPE2 cold wave events. The other two events are studied separately.

**Figure 7:**
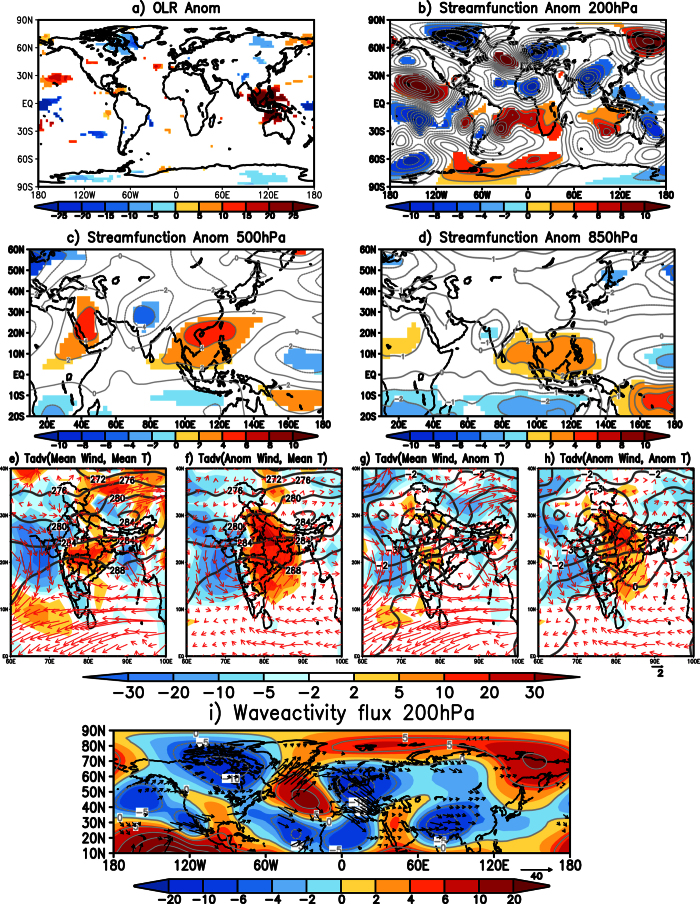
Same as Fig. 2 but for TYPE2 events. (Figure was created using a free software Grid Analysis and Display System (GrADS) version 2.1.a3 (http://cola.gmu.edu/grads/downloads.php)).

Composite of OLR anomalies (Fig. 7a) shows negative anomalies in the equatorial central Pacific due to warmer SST anomalies in the region (Fig. 1c) and positive OLR anomalies over west Pacific (Fig. 7a). The associated eddy streamfunction anomaly at 200 hPa shows cyclonic anomalies covering whole of the Indian subcontinent and parts of China (Fig. 7b). The cyclonic anomaly over the Indo-China region is seen extending to lower levels and is also seen at 500 hPa (Fig. 7c) and 850 hPa (Fig. 7d), similar to the often-observed extension of upper level troughs associated with western disturbances as closed lows in the lower troposphere^2^,^3^,^4^. At 850 hPa (Fig. 7d), the cyclonic anomaly is seen confined to the west coast of India. The other significant feature of the eddy streamfunction anomaly at 500 hPa and 850 hPa is a pair of anticyclones over west Pacific, which is the well-known Matsuno-Gill response to the equatorial west Pacific SST anomalies. The major difference between TYPE1 and TYPE2 events is the barotropic pattern of cyclonic anomalies over India in TYPE2 (in TYPE1, it is baroclinic). Therefore, at low-levels, different processes appear to force the cyclonic anomalies over India in TYPE1 and TYPE2 events.

To see whether the cyclonic anomaly at 200 hPa (Fig. 7b) was a response to the Pacific SST anomalies during the event, we calculated waveactivity flux diagnostic at 200 hPa. Waveactivity flux shows anomalous flux from Pacific to indirectly contribute to the anomalous cyclonic anomaly over the Indian latitudes (Fig. 7i). The flux from the southern parts of North America is seen maintaining the anomalous anticyclone over the North Atlantic. The waveactivity flux from the North Atlantic anomalous anticyclone is seen contributing to the anomalous cyclonic anomalies over India (Fig. 7i). Therefore, the cyclonic anomaly over the Indian latitudes is indirectly linked to the Pacific due to the anomalous response and chain reactions to the Pacific SST anomalies during the events.

We calculated the horizontal advection of mean and anomalous temperature by mean and anomalous winds at 850 hPa to understand the processes maintaining the TYPE2 cold waves over India. The results are shown in Fig. 7e–h. During the TYPE2 cold wave events, north-westerly winds are seen advecting cold temperatures into the north-western parts of India causing cold temperatures and southerly winds are seen advecting warm temperatures from the equatorial region into the central and northern parts of India causing warm temperatures over the region (Fig. 7e). The western flank of the low level anomalous cyclone over the west coast of India, a response to the Pacific convection anomalies, advects cooler temperatures into northwest India from higher latitudes (Fig. 7f) and the eastern flank of the anomalous cyclonic circulation advects warmer equatorial temperatures into central and northern parts of India (Fig. 7f). The advection of cold temperatures to the northwest of India and warm temperatures to other parts of India is also due to the advection of anomalous temperature by the mean observed and the anomalous winds (Fig. 7g–h). The anticyclonic anomaly over the west Pacific and the Bay of Bengal is also seen contributing to the advection of warm temperatures to the Indian landmass (Fig. 7e–h). The above analysis shows that the maintenance of the TYPE2 cold waves over India and their confinement to the north-western parts of India can largely be explained by the advection of the temperatures from the higher latitudes and equatorial regions by the mean and anomalous winds observed during the TYPE2 cold wave events.

We plotted the composite of 850 hPa eddy streamfunction anomalies from 5 days before (DAY-5) to the day of the events (DAY0) (Fig. 4e–h) to understand the origin of TYPE2 cold waves. On DAY-5, 5 days before the cold wave events were observed over the Indian region, cyclonic anomalies can be seen over parts of China and to the northwest of India (Fig. 4e). The cyclonic anomaly is seen moving eastward to the western parts of India from DAY-5 to DAY0 (Fig. 4e–h). The cyclonic anomaly over west India is seen to persist as a significant low throughout the period of the TYPE2 cold wave event (Fig. 7d). Anomalous anticyclone over the North Atlantic is seen prominently from DAY-5 to DAY0 (Fig. 4e–h). The above analysis shows that the TYPE2 cold waves originate as eastward moving transients in the higher latitudes and are seen persisting over west India as a cyclonic circulation throughout the life of the event.

**Figure 8:**
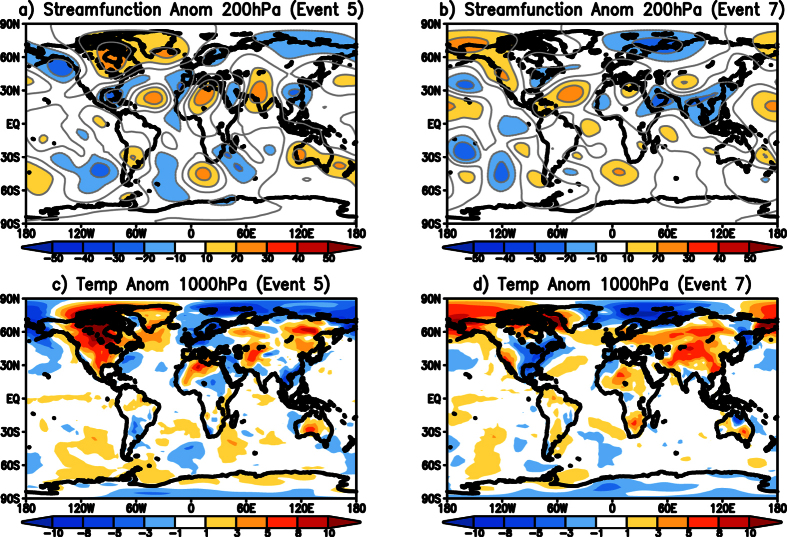
(**a**) Eddy streamfunction (×10^6^ m^2^ s^−1^) anomalies at 200 hPa for the event 5 of TYPE2 (Table 2). (**b**) same as (**a**) but for event 7 of TYPE2 (Table 2). (**c**) same as (**a**) but temperature (°C) anomalies at 1000 hPa. (**d**) same as (**b**) but for temperature anomalies at 1000hPa. (Figure was created using a free software Grid Analysis and Display System (GrADS) version 2.1.a3 (http://cola.gmu.edu/grads/downloads.php)).

Events 5 and 7 (Table 2), which had an opposite phase of eddy streamfunction anomalies at 200 hPa over the Indo-china region compared to the other identified TYPE2 events, are also due to an anomalous quasi-stationary wave from the Pacific to the Indian region (Fig. 8a and b). However, it is seen that during event number 5, the teleconnection caused an anticyclonic anomaly over India and the area of negative temperature anomalies is smaller compared to other events (Fig. 8c). During event 7, anticyclonic anomalies that are to the north of India (Fig. 8b) result in positive temperatures to the north of India (Fig. 8d). Over India, southern parts of India experienced cooler temperatures.

## Discussion

In the present study, we tried to understand the processes causing and maintaining cold waves over India. Based on the standard deviation of the Tmin and following the criteria of IMD, a total of twenty-nine cold wave events were identified. Interestingly, it is found that of the twenty-nine identified events twenty-one events occurred during La Niña years (TYPE1) and eight events during El Niño years (TYPE2) indicating that ENSO provides favorable conditions for the occurrence of cold waves over India. Compositing the events showed that TYPE1 events during La Niña years had a larger spatial extent and caused cooler temperatures over most parts of India whereas TYPE2 events caused cooler temperatures only confined to northwest India.

Analysis of 200 hPa eddy streamfunction anomalies shows the TYPE1 cold waves to be associated with an anomalous wave train from the Ural-Siberia region to South China. The anomalous wave train not necessarily related to La Niña, causes cyclonic anomalies over the Indo-China region causing cooler than normal temperatures over the region. The Matsuno-Gill response to the equatorial convection is such as to cause cyclonic anomalies over Indian region during the cold wave events. A north-south oriented trough covering the whole Indian landmass is seen by the merging of the high latitude cyclonic anomalies with the Matsuno-Gill response. The anomalous north-south oriented trough is seen transporting cold temperatures from higher latitudes into India, resulting in cold waves over India. Therefore, presence of the Matsuno-Gill response to the La Niña type SST anomalies, in association with the anomalous wave train from the Ural-Siberian region, was a key factor for the sustenance of the TYPE1 cold waves.

The TYPE2 cold waves are also seen to have a link to the anomalous conditions in the equatorial Pacific during the events. The anomalous flux from the equatorial Pacific is seen maintaining an anticyclone over the North Atlantic. A wave train from the North Atlantic is then seen causing upper level cyclonic anomaly over the Indian latitudes. The upper level cyclonic anomaly is seen extending to lower levels as a low pressure system over the west coast of India. The low level cyclonic anomaly helps advect cold temperatures from higher latitudes into northwest India causing cold waves over the region. The cyclonic anomaly also advects warm temperatures from lower latitudes into the Indian landmass; thus, confining the cold waves to northwest India.

The identification of the processes causing cold waves, will help in improving their forecast and hence will be beneficial to the society.

## Methods

### Identification of cold wave events

The methodology followed for identifying the cold wave events is similar to that used by the authors for identifying heat wave events over India^15^. In this study, for identifying the cold wave events, we use the high resolution gridded temperature daily minimum temperature dataset of the India Meteorological Department (IMD)^16^. The data are available from 1951 to 2013. We used the data from the satellite era, 1982 to 2013 for identifying cold wave events over India. Daily Tmin anomalies are derived based on daily climatology from 1982 to 2010. For identifying cold waves, Tmin anomalies from 1^st^ November to 28 February over the period 1982 to 2013 are area averaged over the region 71°E-80°E; 21°N-30°N. The area averaged time series is normalized by its own standard deviation. We pick up cold wave events when the normalized area average Tmin anomalies are less than one standard deviation for 4 or more days. Spatial distribution of the Tmin anomalies is checked for all the days of the event to satisfy the criteria suggested by the IMD. The criterion used by IMD^17^ in identifying cold waves is that:

i) Cold waves need not be considered till minimum temperature of a station reaches 10 °C or lower.

ii) When normal minimum temperature of a station is more than or equal to 10 °C: A departure of 5 °C to 6 °C from normal is to be considered a cold wave.

iii) When the normal minimum temperature of a station is less than 10 °C: A departure of 3 °C to 4 °C from normal is to be considered a cold wave.

EOF analysis is carried in the study on the daily Tmin anomalies from 1^st^ November to 28^th^ February over the period 1982 to 2013, covering the region 70°E-90°E; 12°N-30°N.

The European Center for Medium range Weather Forecast (ECMWF) Interim (ERA Interim) reanalysis^18^ dataset at 2.5° × 2.5° horizontal resolution is used to diagnose various mechanism and processes of the cold waves. Daily anomalies of various fields are derived from the daily climatology based on 1982–2010 period. The daily anomalies corresponding to the cold wave events are composited and the significance of the composites is tested using two-tailed Student’s t-test^19^,^20^.

## Additional Information

**How to cite this article**: Ratnam, J. V. *et al*. ENSO’s far reaching connection to Indian cold waves. *Sci. Rep.* **6**, 37657; doi: 10.1038/srep37657 (2016).

**Publisher’s note:** Springer Nature remains neutral with regard to jurisdictional claims in published maps and institutional affiliations.

## Figures and Tables

**Table 1:** List of TYPE1 cold wave events Over India.

TYPE1 events		
1) 8–17 Nov 1983	8) 9–14 Jan 1989	15) 21–25 Jan 2008
2) 27–30 Jan 1984	9) 19–23 Feb 1989	16) 30 Jan-2 Feb 2008
3) 5–9 Feb 1984	10) 9–12 Feb 1989	17) 7–15 Feb 2008
4) 20–28 Feb 1984	11) 8–13 Dec 1996	18) 4–12 Jan 2011
5) 19–23 Dec 1984	12) 9–12 Jan 1999	19) 10–14 Jan 2012
6) 11–18 Feb 1985	13) 11–15 Dec 2005	20) 8–11 Feb 2012
7) 4–8 Jan 1986	14) 6–10 Jan 2006	21) 4–9 Jan 2013

**Table 2:** List of TYPE2 cold wave events over India.

TYPE2 events		
1) 11–15 Jan 1983	4) 19–25 Dec 1986	7) 14–19 Jan 2003
2) 3–8 Feb 1983	5) 6–9 Dec 1987	8) 19–23 Feb 2005
3) 14–17 Dec 1986	6) 31 Dec 1990–6 Jan 1991	
